# Screen time and problem behaviors in children: exploring the mediating role of sleep duration

**DOI:** 10.1186/s12966-019-0862-x

**Published:** 2019-11-14

**Authors:** Michelle D. Guerrero, Joel D. Barnes, Jean-Philippe Chaput, Mark S. Tremblay

**Affiliations:** 1 Healthy Active Living and Obesity Research Group, Children’s Hospital of Eastern Ontario Research Institute, 401 Smyth Road, Ottawa, ON K1H 8L1 Canada; 2 Department of Pediatrics, University of Ottawa, Ottawa, ON K1N 6N5 Canada

**Keywords:** Television/movies, Video games, Mature-rated video games, Negative binomial structural equation modeling, Aggressive behavior, Rule-breaking behavior

## Abstract

**Background:**

Previous research examining the relationship between screen time (ST) and psychological health outcomes have primarily focused on one type of ST (i.e., television), while little research has considered other types of screens (e.g., videos, movies, social media), screen content (e.g., violent video games), or potential mediating variables. Therefore, the purpose of the present study was to assess ST types and content and their association with problem behaviors, and to determine whether these relationships were mediated by sleep duration.

**Methods:**

Parents and children provided cross-sectional baseline data (2016–18) as part of the Adolescent Brain Cognitive Development study, a broadly US representative sample of 11,875 children aged 9 to 10 years. Parents self-reported their children’s emotional and behavioral syndromes via the Child Behavior Checklist and sleep duration using one item from the Parent Sleep Disturbance Scale. Children self-reported their ST behavior, which comprised ST types (television/movies, videos, video games, and social media) and content (mature-rated video games and R-rated movies).

**Results:**

Time spent in various ST types was positively associated with problem behaviors: watching television/movies was associated with a 5.9% increase in rule-breaking behavior (incidence rate ratio [IRR] = 1.059), 5% increase in social problems (IRR = 1.050), 4% increase in aggressive behavior (IRR = 1.040), and 3.7% increase in thought problems (IRR = 1.037). Greater time spent playing mature-rated video games was associated with greater somatic complaints (IRR = 1.041), aggressive behavior (IRR = 1.039), and reduced sleep duration (IRR = .938). Sleep duration mediated the relationship between ST (type and content) and problem behaviors, albeit the effect sizes were small. The largest effects were observed between sleep duration and all problem behaviors, with greater sleep duration predicting an 8.8–16.6% decrease in problem behaviors (IRRs ranging from .834 to .905).

**Conclusion:**

Greater time spent in ST behavior was associated with greater problem behaviors among children. There was strong evidence that longer sleep duration was associated with reduced problem behaviors. While sleep duration mediated the effects of ST on problem behaviors, other potential mediating variables need to be investigated in future research.

## Introduction

Children and youth’s time spent in front of screens – such as televisions, computers, tablets, gaming consoles, and smartphones – continues to increase [1]. This pervasive sedentary behavior has raised concerns among parents, health care professionals, educators, and researchers about the effects of screen time on young people’s well-being. Increased screen time (ST) has been linked with unfavorable body composition, higher cardiometabolic risk, unfavorable behavioral conduct, lower fitness, and lower self-esteem in children [2, 3]. Considering this evidence, expert groups (e.g., the Canadian Society for Exercise Physiology) [4] have issued guidelines on recreational ST that recommend no more than 2 h per day for children and youth (5–17 years), no more than 1 h for preschoolers (3–4 years) and older toddlers (2–3 years), and avoiding all screens for young toddlers and infants (< 2 years).

Previous studies have examined associations between ST and a broad array of psychological health indicators (e.g., anxiety, depression, aggression, attention problems) among children and youth, yet results from these works have yielded mixed findings. In a recent systematic review of reviews, [5] moderately strong evidence was found for associations between ST and depressive symptoms and weak evidence for associations of ST with problem behaviors, anxiety, hyperactivity, inattention, and poor sleep. Conclusions of this review highlight that a major limitation of the existing research is the primary focus on one type of ST – television watching – and that very little is known regarding the mechanisms by which ST is related to psychological health indicators.

Screen-based technology is rapidly evolving, with children and youth frequently engaging with different types of screens and exploring diverse content. Thus, it is imperative that researchers acknowledge this landscape by examining how the different types of screens and screen content relate to emotional and behavioral health indicators. Additionally, several researchers have called for more studies examining potential mediators for the relationship between ST and psychological health outcomes [5, 6]. One potential explanation is that time spent engaging in ST might replace time spent sleeping. Few studies have tested this proposal. In two independent studies with adolescents (15-year-olds), sleep duration mediated the relationship between computer use and health symptoms (e.g., nervousness, headache), [7] and sleep onset difficulties mediated the relationship between computer use and psychological symptoms (e.g., feeling low, irritability) [8]. Other research has shown that adolescents’ (11- to 15-year-olds) sleep onset difficulties and sleep duration mediated the relationship between ST and psychological distress [9]. While these studies contribute to our understanding of potential mechanisms, their findings are limited to adolescents and ignore the different types of ST and ST content. Therefore, the purpose of this study was to examine the associations between ST (types and content) and problem behaviors among children (9–10 years old), and to determine whether these associations were mediated by sleep duration. ST types included television/movies, videos, video games, and social media (text, video chat, and social networking sites), and ST content included mature-rated video games and R-rated movies.

## Methods

### Study population

We used baseline (cross-sectional) data (2016–18) from the Adolescent Brain Cognitive Development (ABCD) study, a US representative sample of 11,875 children aged 9 to 10 years [10]. The ABCD study is an ongoing longitudinal, observational study exploring the development and health among children from age 9 years through early adulthood, with a focus on brain health and cognition. Data for this study will be collected on a biennial-to-annual basis over a 10-year period across 21 sites throughout the United States. Details on the sample, recruitment procedures, measures, and compensation are available elsewhere [11, 12]. Ethics approval was obtained from all relevant institutional research ethics boards as well as signed informed consent from parents/guardians and assent from participating children.

### Exposures

Sleep duration was assessed using one item from the Parent Sleep Disturbance Scale for Children [13]. Parents responded to the following question: “How many hours of sleep does your child get on most nights?” Recreational ST was measured using the Youth Screen Time Survey (14 items) [14]. Children were asked to report the number of hours spent on a typical weekday and weekend day for the following six ST types: watching television/movies, watching videos (e.g., YouTube), playing video games, texting, visiting social networking sites (e.g., Twitter, Instagram), and using video chat (e.g., FaceTime). Children responded to each question using a 7-point scale: none, < 30 min, 30 min, 1 h, 2 h, 3 h, or ≥ 4 h. Texting, using social networking sites, and using video chat were collapsed due to low use of these behaviors and collectively labelled as social media. Daily recreational ST for all six behaviors was calculated by taking a weighted average of the weekday and weekend ST activity (sum of weekday ST behavior [e.g., television] in decimal hours × 5) + (sum of weekend day ST behavior [e.g., television] in decimal hours × 2) / 7. ST behaviors were then categorized into four different groups: < 1 h, 1-1.99h, 2-2.99h, and ≥ 3 h for analysis. ST content was assessed with two items, whereby youth were asked to report how often they played mature-rated video games (e.g., Call of Duty) and watch R-rated movies. Response categories for these questions were scored on a 4-point scale: never, once in a while, regularly, and all the time.

### Outcomes

The Child Behavior Checklist (CBCL) is a parent-report measure used to assess a broad range of emotional and behavioral syndromes among children aged 6 to 18 years [11, 15]. The CBCL comprises eight syndrome scales: anxious/depressed (e.g., “fears doing bad”), withdrawn/depressed (e.g., “rather be alone”), somatic complaints (e.g., “nightmares”), social problems (e.g., “unliked”), thought problems (e.g., “hears things”), attention problems (e.g., “acts too young”), rule-breaking behavior (e.g., “lacks guilt”), and aggressive behavior (e.g., “attacks people”). Respondents answered questions on a 3-point scale: not true, somewhat/sometimes true, or very true/often true. We followed the recommendations of Achenbach and Rescorla [15] and used raw scores (vs. standardized scores) in all data analyses. Scores on the CBCL have shown adequate validity and reliability (α = .78 to .94) [15], and the 8-syndrome has displayed strong fit indices in 30 different societies (e.g., Root Mean Square Error of Approximation were < .06 for all samples) [16].

### Data analytic plan

Missing values (< 1%) were replaced using multiple imputation. Study hypotheses were tested using *M*plus Version 8.2 [17]. The CBCL syndrome scales were treated as count variables. Because the CBCL syndrome scales displayed overdispersion (i.e., greater variances than means), we applied a negative binomial distribution. The model was estimated using maximum likelihood with robust standard errors (MLR) estimation as well the TYPE = COMPLEX function to account for non-independence of observations. Traditional absolute indices of fit are not provided as *M*plus does not generate these indices for models with count variables. We controlled for sex, ethnicity, parental education, family household income, body mass index, and physical activity (Additional file 1: Material S1). Using the MODEL INDIRECT command in *M*plus, we examined the mediating role of sleep duration by testing all possible indirect pathways between each ST behavior (type and content) and behavioral syndrome. Bootstrapping was used to test the significance of the mediation/indirect effects. Given that bootstrapping is not available for models estimated in Mplus 8.2 with the TYPE = COMPLEX option, we used bootstrapping (B = 5000) to obtain 95% confidence intervals without consideration of the nested structure of the data. Incidence rate ratios (IRRs), the exponentiated *B* values, were calculated as an estimate of effect size.

## Results

Means, standard deviations, and frequencies for all study variables are presented in Table 1. The most frequently used ST type was television/movies (approximately 1.25 h/day) and the least frequently used ST type was social media (approximately 0.5 h/day). Aggressive behavior, attention problems, and anxious/depressed were the most frequently reported problem behaviors.

**Table 1:** Descriptives statistics

Variables	Value
Age, years (*n* = 11,875)	9.91 (.62)
Sex (*n* = 11,869)	
Female	5681
Male	6188
Parental income^a^ (*n* = 10,857): *M* (SD)	7.22 (2.42)
Parental education^b^ (*n* = 11,858): *M* (SD)	17.06 (2.67)
Ethnicity (*n* = 11,755)	
White	6176
African American	1779
Hispanic	2047
Asian	255
Multiracial	1498
Body mass index^c^ (*n* = 11,832): *M* (SD)	18.80 (4.17)
Physical activity, active days/week (*n* = 11,844): *M* (SD)	3.49 (2.32)
Screen time types, hours/day: *M* (SD)	
Television/movies (*n* = 11,841)	1.26 (1.04)
Videos (*n* = 11,841)	.98 (1.13)
Video games (*n* = 11,838)	1.01 (1.10)
Social media (*n* = 11,769)	.54 (1.15)
Text	.22 (.54)
Social networking sites	.12 (.42)
Video chat	.19 (.49)
Mature-rated video games (*n* = 11,850)	.38 (.64)
R-rated movies (*n* = 11,850)	.56 (.87)
Problem behaviors (*n* = 11,864): *M* (SD)	
Anxious/depressed (range 0–26)	2.52 (3.01)
Withdrawn/depressed (range 0–15)	1.03 (1.71)
Somatic complaints (range 0–16)	1.49 (1.95)
Social problems (range 0–18)	1.62 (2.27)
Thought problems (range 0–18)	1.62 (2.19)
Attention problems (range 0–20)	2.98 (3.49)
Rule-breaking behavior (range 0–20)	1.19 (1.86)
Aggressive behavior (range 0–36)	3.26 (4.35)
Sleep duration, hours (*n* = 11,869): *M* (SD)	9.00 (1.11)

*Note. M*  means; SD  standard deviation; ST. ^a^Combined income in past 12 months from all sources before taxes and deductions on a scale of 1 = < $5000; 2 = $5000–$11,999; 3 = $12,000–$15,999; 4 = $16,000–$24,999; 5 = $25,000–$34,999; 6 = $35,000–$49,999; 7 = $50,000–$74,999; 8 = $75,000–$99,999; 9 = $100,000–$199,999; 10 = > $200,000. ^b^Highest score on a scale of 0 = Never attended/Kindergarten; 1 = 1st grade; 2 = 2nd grade; 3 = 3rd grade; 4 = 4th grade; 5 = 5th grade; 6 = 6th grade; 7 = 7th grade; 8 = 8th grade; 9 = 9th grade; 10 = 10th grade; 11 = 11th grade; 12 = 12th grade; 13 = High school graduate; 14 = GED or equivalent Diploma; 15 = Some college; 16 = Associate degree, Occupational; 17 = Associate degree, Academic Program; 18 = Bachelor’s degree; 19 = Master’s degree; 20 = Professional School degree; 21 = Doctoral degree. ^c^Body mass index = kg/m^2^

**Fig. 1:**
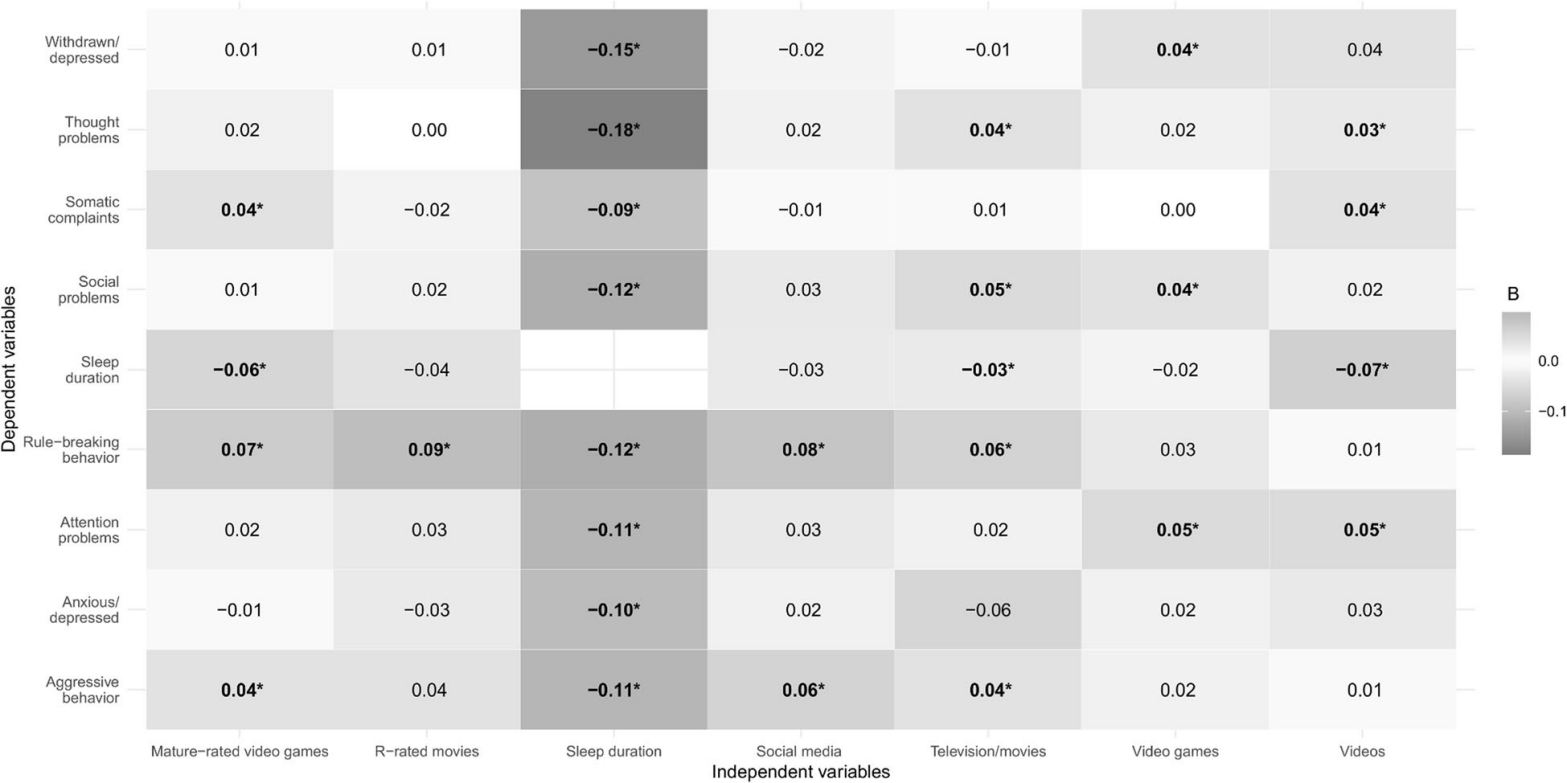
Direct effects between independent variables (screen time types, screen time content, and sleep duration) and problem behaviors. Values represent unstandardized beta coefficients

The direct effects of ST (types and content) and sleep duration on problem behaviors are displayed in Table 2 and Fig. 1. Most of the direct effects were not significant. As depicted in Fig. 1, sleep duration was significantly associated with all eight problem behaviors, suggesting that every one hour increase in sleep duration was associated with an 8.8–16.6% decrease in problem behaviors (IRRs ranging from .912 to .834). Furthermore, every one hour increase in watching television/movies was related to a 5.9% increase in rule-breaking behavior (IRR = 1.059), 5% increase in social problems (IRR = 1.050), 4% increase in aggressive behavior (IRR = 1.040), and 3.7% increase in thought problems (IRR = 1.037).

**Table 2:** Direct effects of screen time behavior and sleep duration

	*B*	*p*	95% CI	IRR	Change per count (%)
Anxious/depressed					
Television/movies	−.01	.623	−.028, .017	.994	
Videos	.03	.136	−.008, .061	1.027	
Video games	.02	.312	−.014, .045	1.015	
Social media	.02	.291	−.013, .044	1.015	
Mature-rated video games	−.01	.769	−.049, .036	1.006	
R-rated movies	−.03	.132	−.072, .009	.994	
Sleep duration	**−.10**	**<.001**	**−.123, −.077**	**.905**	**−9.5%**
Withdrawn/depressed					
Television/movies	−.01	.696	−.029, .019	.969	
Videos	.04	.065	−.002, .071	1.035	
Video games	**.04**	**.034**	**.002, .060**	**1.031**	**3.1%**
Social media	−.02	.558	−.065, .035	.985	
Mature-rated video games	.01	.792	−.032, .043	1.005	
R-rated movies	.01	.695	−.048, .072	1.012	
Sleep duration	**−.15**	**<.001**	**−.172, −.130**	**.860**	**−14%**
Somatic complaints					
Television/movies	.01	.305	−.010, .031	1.011	
Videos	**.04**	**.006**	**.012, .073**	**1.044**	**4.4%**
Video games	.00	.815	−.020, .026	1.003	
Social media	−.01	.716	−.042, .029	.993	
Mature-rated video games	**.04**	**.009**	**.010, .071**	**1.041**	**4.1%**
R-rated movies	−.02	.542	−.066, .035	.985	
Sleep duration	**−.09**	**<.001**	**−.117, −.068**	**.912**	**−8.8%**
Social problems					
Television/movies	**.05**	**<.001**	**.024, .073**	**1.050**	**5%**
Videos	.02	.180	−.010, .051	1.021	
Video games	**.04**	**.026**	**.005, .074**	**1.040**	**4%**
Social media	.03	.114	−.008, .073	1.033	
Mature-rated video games	.01	.661	−.022, .035	1.006	
R-rated movies	.02	.226	−.013, .055	1.021	
Sleep duration	**−.12**	**<.001**	**−.142, −.097**	**.888**	**−11.2%**
Thought problems					
Television/movies	**.04**	**<.001**	**.019, .054**	**1.037**	**3.7%**
Videos	**.03**	**.022**	**.005, .059**	**1.033**	**3.3%**
Video games	.02	.066	−.002, .048	1.023	
Social media	.02	.252	−.017, .066	1.025	
Mature-rated video games	.02	.439	−.023, .054	1.015	
R-rated movies	−.00	.802	−.042, .034	.996	
Sleep duration	**−.18**	**<.001**	−.202, −.161	**.834**	**−16.6%**
Attention problems					
Television/movies	.02	.062	−.001, .039	1.019	
Videos	**.05**	**.001**	**.018, .072**	**1.046**	**4.6%**
Video games	**.05**	**<.001**	**.026, .069**	**1.049**	**4.9%**
Social media	.03	.091	−.005, .071	1.033	
Mature-rated video games	.02	.107	−.005, .054	1.024	
R-rated movies	.03	.146	−.010, .069	1.030	
Sleep duration	**−.11**	**<.001**	**−.131, −.090**	**.895**	**−10.5%**
Rule-breaking behavior					
Television/movies	**.06**	**<.001**	**.025, .090**	**1.059**	**5.9%**
Videos	.01	.391	−.017, .044	1.013	
Video games	.03	.115	−.006, .057	1.026	
Social media	**.08**	**<.001**	**.046, .115**	**1.084**	**8.4%**
Mature-rated video games	**.07**	**<.001**	**.046, .101**	**1.076**	**7.6%**
R-rated movies	**.09**	**<.001**	**.038, .132**	**1.089**	**8.9%**
Sleep duration	**−.12**	**<.001**	**−.144, −.091**	**.889**	**−11.1%**
Aggressive behavior					
Television/movies	**.04**	**.001**	**.015, .063**	**1.040**	**4%**
Videos	.01	.561	−.019, .034	1.008	
Video games	.02	.145	−.008, .053	1.023	
Social media	**.06**	**<.001**	**.029, .053**	**1.062**	**6.2%**
Mature-rated video games	**.04**	**.031**	**.003, .092**	**1.039**	**3.9%**
R-rated movies	.04	.117	−.009, .073	1.038	
Sleep duration	**−.11**	**<.001**	**−.136, −.083**	**.896**	**−10.4**
Sleep duration					
Television/movies	**−.03**	**.017**	−.005, −.006	**.970**	**−3%**
Videos	**−.07**	**<.001**	**−.101, −.044**	**.930**	**−7%**
Video games	−.02	.096	−.032, .003	.986	
Social media	−.03	.064	−.068, .002	.968	
Mature-rated video games	**−.06**	**<.001**	**−.098, −.031**	**.938**	**−6.2%**
R-rated movies	−.04	.139	**−.082, .011**	.965	

*Note.* *CI*  confidence intervals; *IRR*  incidence rate ratios. Adjusted for sex, parental education, family income, ethnicity, physical activity, and body mass index.

Relationships with p-values less than or equal to 0.05 and confidence intervals excluding zero are in bold font

The indirect effects of ST behaviors were small (see Table 3). Overall, the patterns of these results suggest that greater engagement in ST types and content was associated with shorter sleep duration, which in turn was associated with increased problem behaviors. The indirect effects of watching videos produced the largest effect; every one hour increase in watching videos was associated with reduced sleep duration, which consequently was related to a 1.7% increase in anxious/depressed, 1.3% increase in thought problems, and 1.1% increase in withdrawn/depressed.

**Table 3:** Indirect effects of screen time behaviors through sleep

	*B*	*p*	95% CI	IRR	Change per count (%)
Television/movies → anxious/depressed	**.00**	**.005**	**.001, .005**	**1.003**	**.3%**
Videos → anxious/depressed	**.01**	**<.001**	**.005, .010**	**1.017**	**1.7%**
Video games → anxious/depressed	.00	.241	−.001, .004	1.001	
Social media → anxious/depressed	.00	.059	.000, .007	1.003	
Mature-rated video games → anxious/depressed	**.01**	**<.001**	**.003, .010**	**1.006**	**.6%**
R-rated movies → anxious/depressed	**.00**	**.038**	**.000, .007**	**1.004**	**.4%**
Television/movies → withdrawn/depressed	**.01**	**.005**	**.002, .008**	**1.005**	**.5%**
Videos → withdrawn/depressed	**.01**	**<.001**	**.007, .015**	**1.011**	**1.1%**
Video games → withdrawn/depressed	.00	.238	−.001, .006	1.002	
Social media → withdrawn/depressed	.01	.059	.000, .010	1.005	
Mature-rated video games → withdrawn/depressed	**.01**	**<.001**	**.005, .015**	**1.010**	**1%**
R-rated movies → withdrawn/depressed	**.01**	**.036**	**.000, .011**	**.998**	**↓.2%**
Television/movies → somatic complaints	**.00**	**.006**	**.001, .005**	**1.003**	**.3%**
Videos → somatic complaints	**.01**	**<.001**	**.004, .010**	**1.007**	**.7%**
Video games → somatic complaints	.00	.242	−.001, .004	1.001	
Social media → somatic complaints	.00	.065	.000, .006	1.003	
Mature-rated video games → somatic complaints	**.01**	**<.001**	**.003, .009**	**1.006**	**.6%**
R-rated movies → somatic complaints	**−.00**	**.039**	**.000, .007**	**1.001**	**.1%**
Television/movies → social problems	**.00**	**.004**	**.001, .006**	**1.004**	**.4%**
Videos → social problems	**.01**	**<.001**	**.006, .011**	**1.009**	**.9%**
Video games → social problems	.00	.238	−.001, .005	1.002	
Social media → social problems	.00	.058	.000, .008	1.004	
Mature-rated video games→ social problems	**.01**	**<.001**	**.004, .012**	**1.008**	**.8%**
R-rated movies → social problems	**.00**	**.038**	**.000, .008**	**.998**	**↓.2%**
Television/movies → thought problems	**.01**	**.004**	**.002, .009**	**1.006**	**.6%**
Videos → thought problems	**.01**	**<.001**	**.009, .018**	**1.013**	**1.3%**
Video games → thought problems	.00	.236	−.002, .007	1.003	
Social media → thought problems	.01	.057	.000, .012	1.006	
Mature-rated video games → thought problems	**.01**	**<.001**	**.007, .017**	**.006**	**.6%**
R-rated movies → thought problems	**.01**	**.035**	**.001, .012**	**1.001**	**.1%**
Television/movies → attention problems	**.00**	**.004**	**.001, .006**	**1.003**	**.3%**
Videos → attention problems	**.01**	**<.001**	**.005, .011**	**1.008**	**.8%**
Video games → attention problems	.00	.239	−.001, .004	1.002	
Social media → attention problems	.00	.058	.000, .007	1.004	
Mature-rated video games→ attention problems	**.01**	**<.001**	**.004, .011**	**1.007**	**.7%**
R-rated movies → attention problems	**.00**	**.037**	**.000, .008**	**.997**	**↓.3%**
Television/movies → rule-breaking behavior	**.00**	**.005**	**.001, .006**	**1.004**	**.4%**
Videos → rule-breaking behavior	**.01**	**<.001**	**.006, .012**	**1.009**	**.9%**
Video games → rule-breaking behavior	.00	.240	−.001, .005	1.002	
Social media → rule-breaking behavior	.00	.060	.000, .008	1.004	
Mature-rated video games → rule-breaking behavior	**.01**	**<.001**	**.004, .012**	**1.008**	**.8%**
R-rated movies → rule-breaking behavior	**.00**	**.038**	**.000, .008**	**.990**	**↓1%**
Television/movies → aggressive behavior	**.00**	**.005**	**.001, .006**	**1.003**	**.3%**
Videos → aggressive behavior	**.01**	**<.001**	**.005, .011**	**1.008**	**.8%**
Video games → aggressive behavior	.00	.238	−.001, .004	1.002	
Social media → aggressive behavior	.00	.059	.000, .007	1.004	
Mature-rated video games → aggressive behavior	**.01**	**<.001**	**.004, .011**	**1.007**	**.7%**
R-rated movies → aggressive behavior	**.00**	**.037**	**.000, .008**	**.996**	**↓.4%**

*Note.* *CI*  confidence intervals; *IRR*  incidence rate ratios. Adjusted for sex, parental education, family income, ethnicity, physical activity, and body mass index.

Relationships with p-values less than or equal to 0.05 and confidence intervals excluding zero are in bold font

## Discussion

The purpose of this study was to examine the associations between ST (types and content) and problem behaviors among children (9–10 years old), and to determine whether these associations were mediated by sleep duration. Results showed that specific ST types and content were positively (adversely) related to children’s problem behaviors. Sleep duration emerged as a significant mediator, though the effect sizes were small. There was strong evidence that longer sleep duration was associated with fewer problem behaviors.

Several direct relationships were found that warrant discussion. ST types were related to increased problem behaviors, albeit no apparent pattern was identified. Television/movie viewing was associated with four of the eight problem behaviors, whereby greater time spent watching television/movies was associated with increased occurrences of social problems, thought problems, rule-breaking behavior, and aggressive behavior. Consistent with findings from Stiglic and Viner’s [5] systematic review on ST and health outcomes among children and adolescents, our results revealed that television/movie viewing was not associated with attention problems. Our findings also showed that none of the four ST types were related to anxious/depressed syndrome. Playing video games was the only ST type that was associated with withdrawn/depressed syndrome, which is consistent with the notion that more time spent playing video games may be linked with social withdrawal, social isolation, and more internalizing problems. Previous studies examining the relationships between ST and anxiety and depression among children have found that ST was positively associated with anxiety and depression, [18–20] while others have found null findings or even favorable associations of greater ST. [21–23] While our findings on ST and anxiety/depression align with other cross-sectional research documenting null effects, future releases of the ABCD data set will allow researchers to explore longitudinal relations between ST types and problem behaviors. Furthermore, watching television/movies, viewing videos, and playing mature-rated video games were all associated with reduced sleep duration. This finding may be partially explained through the displacement hypothesis. As children spend significant time in ST behaviors, it replaces time given to other activities, such as sleep. Furthermore, when children spend time on screens (especially at night) they are exposed to the blue light, which has been shown to delay sleep onset and reduce sleep quality [24]. Another possible explanation for this finding is that parents who do not implement rules around their children’s ST use may be less likely to have rules around bedtime rules and routines.

We found that greater time spent playing mature-rated video games was associated with greater somatic complaints, aggressive behavior, and reduced sleep duration. Results also showed that time spent playing/watching both mature-rated video games and R-rated movies were positively related to rule-breaking behavior. These findings are generally consistent with past meta-analytic research showing links between mature-rated video games and greater deviant behavior (e.g., risky sexual behavior, delinquency behavior, aggressive behavior), [25] and decreased prosocial behavior and empathy [26]. Reasoning for this relationship may lie within the identity and behavioral simulation logic [27]. Video games, especially character-based games, allow the player to transform into a different person (identity simulation) by experiencing thoughts, feelings, and actions practiced in video games that can spread to non-virtual, real world contexts (behavioral simulation). Thus, mature-rated video games may distort children’s sense of self and perception of the real-world. While some researchers continue to debate the meaningfulness of past research on mature-rated video games, we argue that monitoring children’s exposure to such games remains worthy of our attention.

The largest effect sizes were observed between sleep duration and problem behaviors, with longer sleep duration predicting an 8.8–16.6% decrease in problem behaviors. This finding aligns with previous work describing negative associations between shorter sleep duration and higher internalizing and externalizing symptoms [28–30]. More specifically, results of our study showed that greater sleep duration had the largest impact on children’s thought problems. This finding aligns with Pesonen et al.’s [29] work with 8-year-olds wherein short sleep duration (measured via accelerometers) played a significant role in mother-rated child thought problems. Two basic mechanisms have been proposed for why sleep would impact children’s daytime behavior [31]. The first hypothesis assumes that insufficient sleep prevents or reduces essential brain activities necessary for brain maturation, affect regulation, and learning, whereas the second hypothesis assumes that insufficient sleep leads to increases in daytime sleepiness and reduced alertness, which potentially hinders daytime functioning. Regardless of which mechanism is responsible, results suggest that acquiring sufficient sleep appears to play an important role in predicting children’s psychological well-being and behavior.

Our analysis showed that sleep duration was a significant mediator between ST (types and content) and problem behaviors, consistent with previous research documenting the mediating role of sleep between ST and psychological well-being [9, 32]. A practical implication of this finding is that the negative effects of ST may be partly counteracted by acquiring sufficient sleep. The American Academy of Sleep Medicine (AASM) guidelines – endorsed by the American Academy of Pediatrics (AAP) – recommends that children’s bedrooms are free of any screen-based device and that children should not have access to any screen-based device 30 min before bedtime [33]. Parents can help establish healthy screen media habits for children that can facilitate sleep by speaking with their children about the importance of sleep, developing a bedtime that allows for adequate sleep, encouraging children to engage in calming activities (e.g., reading, coloring) in the evening rather than using electronic devices, applying family rules/routines to all children in the household, and developing a predictable bedtime routine (e.g., brush teeth, read a story, lights out) [34]. Given the effect sizes of sleep were very modest, other potential sleep-related mediating variables (e.g., sleep quality, timing, disturbances, and variability) need to be investigated in future research in order to achieve a more complete understanding of mechanisms responsible for the relationship between ST and psychological health indicators.

Limitations of our study must be acknowledged. As with all cross-sectional research, inferences regarding the direction of the relationships among ST, sleep, and problem behaviors cannot be drawn. Just as ST (types and content) and inadequate sleep may elicit problem behaviors, the degree to which a child experiences problem behaviors may also prompt them to engage in more ST behavior and may interfere with sleep. Another limitation of this research is its reliance on parents to report their child’s sleep duration (one-item) and problem behaviors as well as reliance on children to report their ST behavior. It is very possible that children underestimated their time spent on the different screen types or what constitutes as mature-rated video games or R-rated movies. Conducting longitudinal research using objective measures of ST and sleep will confirm/refute the findings of this observational study. It is important to note that we were only able to examine content of video games (mature-rated) and movies (R-rated), and not content of other screen-based media such as videos or social media. As research on ST continues to grow, there is a pressing need to examine health outcomes of time spent on specific platforms (e.g., Instagram, YouTube, Netflix, TikTok) and potentially content of these platforms, though concerns related to ethics, privacy, and confidentially will make this a difficult endeavor. Nevertheless, finding innovative, reliable approaches to objectively measure ST behavior by differentiating time spent on different platforms should be a priority.

The aforementioned limitations are balanced with several strengths. The large representative sample allowed us to build and test a complex mediating model. Furthermore, our research utilized a relatively novel methodological approach – negative binomial SEM – and extends previous research through the inclusion of each syndrome. Including each syndrome in the model, as opposed to using the broadband scales (internalizing and externalizing), allowed us to identify unique associations of ST types and content on specific problem behaviors. We also included several confounding variables in our analyses – including body mass index and physical activity – that have been typically ignored in previous research.

The current literature on the associations between children and youth ST and psychological well-being is somewhat mixed. These mixed findings have led researchers to debate whether guidelines on ST are necessary. It is clear that there is much to be explored in this area and several methodological limitations need to be addressed. However, the implications of our study, coupled with those of other studies, are clear. Those responsible for ensuring the healthy development of children and youth should pay close attention to how much time young people spend on digital screens as well as the type of screen content they are exposed to. In sum, perhaps erring on the side of caution is the most reasonable approach to the current ST debate [35].

## Supplementary information

**Additional file 1.** Associations between covariates, sleep, problem behaviors

## Data Availability

The data that support the findings of this study are available from the ABCD Study but restrictions apply to the availability of these data, which were used under license for the current study, and so are not publicly available. Data are however available from the authors upon reasonable request and with permission of the ABCD Study.

## A**bbreviations**

ABCD: Adolescent Brain Cognitive Development
CBCL: Child Behavior Checklist
IRR: incidence rate ratio
ST: screen time
