# Supplementary material for: Screen time and problem behaviors in children: exploring the mediating role of sleep duration
Source: Int J Behav Nutr Phys Act. 2019 Nov 14;16:105. doi: 10.1186/s12966-019-0862-x (PMC6854622; doi:10.1186/s12966-019-0862-x)
Supplement: Supplementary file 1 — Additional file 1. Associations between covariates, sleep, problem behaviors [file 12966_2019_862_MOESM1_ESM.docx]

| **Supplementary Material 1. Associations between covariates, sleep, problem behaviors** | | | |
| --- | --- | --- | --- |
|  | B | *p* | 95% CI |
| Anxious/depressed |  |  |  |
| Female (reference: male) | .00 | .961 | -.062, .065 |
| Parental education | **.06** | **.005** | **.018, .101** |
| Family income | **-.11** | **<.001** | **-.153, -.067** |
| Ethnicity (reference: Asian) |  |  |  |
| African American | -.06 | .623 | -.287, .172 |
| Caucasian | **.37** | **.005** | **.108, .624** |
| Hispanic | **.28** | **.027** | **.032, .525** |
| Multiracial | **.36** | **.002** | **.128, .591** |
| Physical activity | -.04 | .371 | -.115, .043 |
| Body mass index | -.00 | .634 | -.005, .009 |
| Withdrawn/depressed |  |  |  |
| Female (reference: male) | **.15** | **<.001** | **.102, .202** |
| Parental education | **-.05** | **.014** | **-.096, -.011** |
| Family income | **-.21** | **<.001** | **-.257, -.155** |
| Ethnicity (reference: Asian) |  |  |  |
| African American | -.21 | .199 | -.527, .110 |
| Caucasian | .10 | .517 | -.204, .406 |
| Hispanic | -.03 | .848 | -.339, .278 |
| Multiracial | .14 | .353 | -.154, .432 |
| Physical activity | **-.19** | **<.001** | **-.284, -.103** |
| Body mass index | **.01** | **.006** | **.003, .019** |
| Somatic complaints |  |  |  |
| Female (reference: male) | **-.13** | **<.001** | **-.174, -.076** |
| Parental education | -.03 | .186 | -.078, .015 |
| Family income | **-.09** | **<.001** | **-.128, -.057** |
| Ethnicity (reference: Asian) |  |  |  |
| African American | **-.15** | **.027** | **-.284, -.017** |
| Caucasian | **.22** | **.010** | **.053, .392** |
| Hispanic | .10 | .189 | -.047, .239 |
| Multiracial | **.24** | **.003** | **.079, .397** |
| Physical activity | .03 | .206 | -.019, .086 |
| Body mass index | **.01** | **<.001** | **.008, .020** |
| Social problems |  |  |  |
| Female (reference: male) | **.11** | **.016** | **.020, .191** |
| Parental education | **-.08** | **<.001** | **-.125, -.035** |
| Family income | **-.17** | **<.001** | **-.223, -.120** |
| Ethnicity (reference: Asian) |  |  |  |
| African American | .12 | .399 | -.158, .397 |
| Caucasian | .27 | .080 | -.032, .562 |
| Hispanic | .16 | .280 | -.129, .446 |
| Multiracial | **.35** | **.014** | **.072, .629** |
| Physical activity | -.01 | .727 | -.080, .056 |
| Body mass index | **.01** | **.001** | **.006, .020** |
| Thought problems |  |  |  |
| Female (reference: male) | **.25** | **<.001** | **.199, .296** |
| Parental education | -.01 | .755 | -.049, .035 |
| Family income | **-.09** | **.001** | **-.145, -.039** |
| Ethnicity (reference: Asian) |  |  |  |
| African American | -.13 | .240 | -.335, .084 |
| Caucasian | **.29** | **.006** | **.084, .491** |
| Hispanic | -.01 | .925 | -.218, .198 |
| Multiracial | **.31** | **.001** | **.123, .503** |
| Physical activity | .01 | .787 | -.061, .081 |
| Body mass index | -.01 | .087 | -.013, .001 |
| Attention problems |  |  |  |
| Female (reference: male) | **.35** | **<.001** | **.276, .418** |
| Parental education | -.03 | .149 | -.062, .009 |
| Family income | **-.09** | **<.001** | **-.132, -.043** |
| Ethnicity (reference: Asian) |  |  |  |
| African American | **.25** | **.001** | **.104, .402** |
| Caucasian | **.34** | **<.001** | **.189, .488** |
| Hispanic | **.26** | **.005** | **.080, .445** |
| Multiracial | **.45** | **<.001** | **.278, .614** |
| Physical activity | .02 | .597 | -.042, .073 |
| Body mass index | **-.01** | **<.001** | **-.016, -.008** |
| Rule-breaking behaviour |  |  |  |
| Female (reference: male) | **.33** | **<.001** | **.271, .396** |
| Parental education | **-.12** | **<.001** | **-.170, -.069** |
| Family income | **-.15** | **<.001** | **-.207, -.088** |
| Ethnicity (reference: Asian) |  |  |  |
| African American | **.37** | **.014** | **.076, .666** |
| Caucasian | **.33** | **.027** | **.038, .624** |
| Hispanic | .19 | .258 | -.139, .519 |
| Multiracial | **.48** | **.002** | **.179, .783** |
| Physical activity | **.09** | **.028** | **.010, .175** |
| Body mass index | -.00 | .686 | -.008, .005 |
| Aggressive behaviour |  |  |  |
| Female (reference: male) | **.23** | **<.001** | **.176, .286** |
| Parental education | -.04 | .065 | -.085, .002 |
| Family income | **-.13** | **<.001** | **-.184, -.066** |
| Ethnicity (reference: Asian) |  |  |  |
| African American | **.31** | **.009** | **.080, .544** |
| Caucasian | **.53** | **<.001** | **.312, .747** |
| Hispanic | **.36** | **.001** | **.142, .577** |
| Multiracial | **.54** | **<.001** | **.339, .743** |
| Physical activity | **.08** | **.006** | **.022, .130** |
| Body mass index | .01 | .110 | -.001, .011 |
| Sleep duration |  |  |  |
| Female (reference: male) | .02 | .405 | -.030, .074 |
| Parental education | **.15** | **<.001** | **.113, .176** |
| Family income | **.14** | **<.001** | **.098, .172** |
| Ethnicity (reference: Asian) |  |  |  |
| African American | **-.46** | **<.001** | **-.601, -.312** |
| Caucasian | **.14** | **.030** | **.014, .271** |
| Hispanic | -.03 | .658 | -.178, .113 |
| Multiracial | -.09 | .223 | -.240, .056 |
| Physical activity | .01 | .733 | -.037, .053 |
| Body mass index | **-.02** | **<.001** | **-.024, -.015** |
